# Response-adapted intensification with cyclophosphamide, bortezomib, and dexamethasone versus no intensification in patients with newly diagnosed multiple myeloma (Myeloma XI): a multicentre, open-label, randomised, phase 3 trial

**DOI:** 10.1016/S2352-3026(19)30167-X

**Published:** 2019-10-14

**Authors:** Graham H Jackson, Faith E Davies, Charlotte Pawlyn, David A Cairns, Alina Striha, Corinne Collett, Anna Waterhouse, John R Jones, Bhuvan Kishore, Mamta Garg, Cathy D Williams, Kamaraj Karunanithi, Jindriska Lindsay, Jamie N Wilson, Matthew W Jenner, Gordon Cook, Martin F Kaiser, Mark T Drayson, Roger G Owen, Nigel H Russell, Walter M Gregory, Gareth J Morgan

**Affiliations:** a Northern Institute for Cancer Research, Newcastle University, Newcastle upon Tyne, UK; b Perlmutter Cancer Center, NY Langone Health, New York, NY, USA; c The Institute of Cancer Research, London, UK; d The Royal Marsden Hospital NHS Foundation Trust, London, UK; e Clinical Trials Research Unit, Leeds Institute of Clinical Trials Research, University of Leeds, Leeds, UK; f Section of Experimental Haematology, Leeds Institute of Cancer and Pathology, University of Leeds, Leeds, UK; g Heart of England NHS Foundation Trust, Birmingham, UK; h Leicester Royal Infirmary, Leicester, UK; i Centre for Clinical Haematology, Nottingham University Hospital, Nottingham, UK; j University Hospital of North Midlands, Stoke-on-Trent, UK; k East Kent Hospitals University NHS Foundation Trust, Canterbury, UK; l Western Sussex Hospitals NHS Foundation Trust, Chichester, UK; m University Hospital Southampton NHS Foundation Trust, Southampton, UK; n Clinical Immunology Service, Institute of Immunology and Immunotherapy, University of Birmingham, Birmingham, UK; o Haematological Malignancy Diagnostic Service, St James's University Hospital, Leeds, UK

## Abstract

**Background:**

Multiple myeloma has been shown to have substantial clonal heterogeneity, suggesting that agents with different mechanisms of action might be required to induce deep responses and improve outcomes. Such agents could be given in combination or in sequence on the basis of previous response. We aimed to assess the clinical value of maximising responses by using therapeutic agents with different modes of action, the use of which is directed by the response to the initial combination therapy. We aimed to assess response-adapted intensification treatment with cyclophosphamide, bortezomib, and dexamethasone (CVD) versus no intensification treatment in patients with newly diagnosed multiple myeloma who had a suboptimal response to initial immunomodulatory triplet treatment which was standard of care in the UK at the time of trial design.

**Methods:**

The Myeloma XI trial was an open-label, randomised, phase 3, adaptive design trial done at 110 National Health Service hospitals in the UK. There were three potential randomisations in the study: induction treatment, intensification treatment, and maintenance treatment. Here, we report the results of the randomisation to intensification treatment. Eligible patients were aged 18 years or older and had symptomatic or non-secretory, newly diagnosed multiple myeloma, had completed their assigned induction therapy as per protocol (cyclophosphamide, thalidomide, and dexamethasone or cyclophosphamide, lenalidomide, and dexamethasone) and achieved a partial or minimal response. For the intensification treatment, patients were randomly assigned (1:1) to cyclophosphamide (500 mg daily orally on days 1, 8, and 15), bortezomib (1·3 mg/m^2^ subcutaneously or intravenously on days 1, 4, 8, and 11), and dexamethasone (20 mg daily orally on days 1, 2, 4, 5, 8, 9, 11, and 12) up to a maximum of eight cycles of 21 days or no treatment. Patients were stratified by allocated induction treatment, response to induction treatment, and centre. The co-primary endpoints were progression-free survival and overall survival, assessed from intensification randomisation to data cutoff, analysed by intention to treat. Safety analysis was per protocol. This study is registered with the ISRCTN registry, number ISRCTN49407852, and clinicaltrialsregister.eu, number 2009–010956–93, and has completed recruitment.

**Findings:**

Between Nov 15, 2010, and July 28, 2016, 583 patients were enrolled to the intensification randomisation, representing 48% of the 1217 patients who achieved partial or minimal response after initial induction therapy. 289 patients were assigned to CVD treatment and 294 patients to no treatment. After a median follow-up of 29·7 months (IQR 17·0–43·5), median progression-free survival was 30 months (95% CI 25–36) with CVD and 20 months (15–28) with no CVD (hazard ratio [HR] 0·60, 95% CI 0·48–0·75, p<0·0001), and 3-year overall survival was 77·3% (95% Cl 71·0–83·5) in the CVD group and 78·5% (72·3–84·6) in the no CVD group (HR 0·98, 95% CI 0·67–1·43, p=0·93). The most common grade 3 or 4 adverse events for patients taking CVD were haematological, including neutropenia (18 [7%] patients), thrombocytopenia (19 [7%] patients), and anaemia (8 [3%] patients). No deaths in the CVD group were deemed treatment related.

**Interpretation:**

Intensification treatment with CVD significantly improved progression-free survival in patients with newly diagnosed multiple myeloma and a suboptimal response to immunomodulatory induction therapy compared with no intensification treatment, but did not improve overall survival. The manageable safety profile of this combination and the encouraging results support further investigation of response-adapted approaches in this setting. The substantial number of patients not entering this trial randomisation following induction therapy, however, might support the use of combination therapies upfront to maximise response and improve outcomes as is now the standard of care in the UK.

**Funding:**

Cancer Research UK, Celgene, Amgen, Merck, Myeloma UK.

## Introduction

The aetiology and progression of multiple myeloma are driven by the accumulation of acquired genetic events that affect clonal competition within the bone marrow microenvironment.^1^, ^2^ Tumour cell diversity increases as genetic lesions accumulate, and the disease progresses from monoclonal gammopathy of undetermined significance to myeloma, leading to substantial subclonal heterogeneity at the time of diagnosis. Applying induction treatment designed to eliminate susceptible clones might provide selective pressure for the expansion of resistant clones, resulting in early or late relapse. Combination chemotherapies designed to maximise tumour cell death and eliminate resistant clones can improve clinical outcomes compared with single-agent chemotherapies. Depth of response has been identified as an independent prognostic factor, making the eradication of minimal residual disease an important therapeutic endpoint.^3^, ^4^, ^5^

Strategies to deepen response after induction therapy include the use of autologous haemopoietic stem cell transplantation (in those eligible) and the use of post-transplant consolidation therapy. Although the optimal timing for achieving maximum response is unclear, in our previous study, Myeloma IX,^6^, ^7^ patients with complete response before transplantation had better progression-free survival and overall survival than patients without complete response, supporting an argument for early achievement of deep responses and the use of pre-transplant intensification rather that post-transplant consolidation.

This association raises the question as to whether monitoring response during induction therapy and switching to an alternative chemotherapy regimen in poor responders could increase the rate and depth of response and improve clinical outcomes. This study is the first to prospectively evaluate such a response-adapted approach to induction therapy for patients with newly diagnosed multiple myeloma. The purpose of the trial was to determine whether treatment intensification with a bortezomib-based regimen improves progression-free survival and overall survival in patients with suboptimal response after immunomodulatory-drug–based induction therapy, which was standard of care in the UK at time of trial design.

**Research in context**

**Evidence before this study**

Potential strategies to deepen response after induction therapy for patients with myeloma include the use of autologous haemopoietic stem cell transplantation (in those eligible) and the use of post-transplant consolidation. Although the optimal timing for achieving maximum response is unclear, we found in our previous study, Myeloma IX, that patients with a complete response before autologous haemopoietic stem cell transplantation had better progression-free and overall survival than patients with a less than complete response. This supports an argument for early achievement of deep responses and the use of pre-transplant intensification rather than post-transplant consolidation. Little data is available concerning strategies for deepening response in transplantation-ineligible patients. We searched PubMed (July 15, 2019) for trials examining a response-adapted intensification strategy, using the search terms “myeloma” and “intensification”, without language restrictions, for clinical trials published before 2019 and excluding those relating to the introduction of autologous haemopoietic stem cell transplantation or those lacking response adaptation. We identified one previous phase 2 study reporting the use of cyclophosphamide, bortezomib and dexamethasone intensification administered to eight patients who failed to achieve more than a partial response to two cycles of cyclophosphamide, thalidomide and dexamethasone induction therapy. Five (63%) of eight deepened their response. To our knowledge the strategy of early response-adapted therapy has not been previously studied in a randomised trial and we sought to investigate this in Myeloma XI.

**Added value of this study**

We found a significant improvement in response and progression-free survival associated with the use of proteasome inhibitor-based intensification therapy for patients who achieved only a minimal or partial response to immunomodulatory triplet induction for newly diagnosed myeloma patients, although no difference in overall survival occurred.

**Implications of all the available evidence**

Our results support the concept that resistance to initial therapy is based on the specific therapy used and can be overcome by switching to a chemotherapy regimen with an alternate mechanism of action. In the event of a suboptimal response to immunomodulatory agent-based induction therapy, switching to a proteasome inhibitor-based combination improved response and progression-free survival. Taken together our data suggest that if agents of several different classes are available and can be tolerated in combination they should be used together upfront as is now the standard of care in the UK. If not, agent class should be switched rapidly in the absence of a deep response with the aim of response intensification to prolong progression-free survival.

## Methods

### Study design and participants

**Figure 1:**
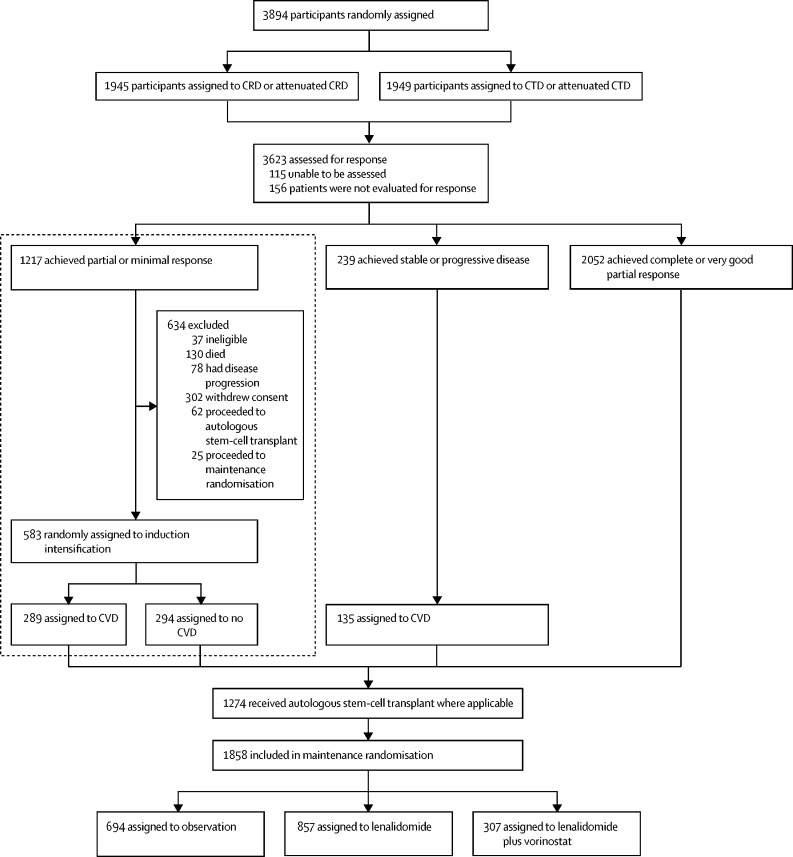
Trial profile of Myeloma XI Area highlighted is the CVD randomisation reported in this manuscript. C=cyclophosphamide. D=dexamethasone. R=lenalidomide. T=thalidomide. V=bortezomib.

The Myeloma XI study was a phase 3, open-label, randomised, adaptive design trial with three randomisation stages (figure 1). There were three potential randomisations in the study: at trial entry for all patients to allocate induction treatment separately for those considered eligible or ineligible for transplantation; after induction treatment for those patients with a suboptimal response to treatment (minimal or partial response based on International Myeloma Working Group response criteria) to allocate induction intensification treatment; and at the completion of induction and intensification or autologous haemopoietic stem cell transplantation (where applicable) to allocate maintenance treatment. This Article reports the results of the randomisation to induction intensification treatment. Results of the induction and maintenance randomisations have been,^8^ or will, be presented elsewhere. The trial recruited from 110 National Health Service hospitals in England, Wales, and Scotland (appendix p 15).

Eligible patients for the overall study were at least 18 years of age and had newly diagnosed multiple myeloma, based on paraprotein in serum or urine, bone marrow clonal plasma cells or plasmacytoma, and myeloma-related symptoms or organ or tissue impairment. Eligible patients for the intensification randomisation reported here additionally had to have completed their assigned induction therapy as per protocol (cyclophosphamide, thalidomide, and dexamethasone or cyclophosphamide, lenalidomide, and dexamethasone) and achieved a partial or minimal response. Patients were excluded at trial entry if they had other previous or concurrent malignancies, including myelodysplastic syndromes, previous treatment for myeloma (excluding local radiotherapy, bisphosphonates, and corticosteroids), grade 2 or higher peripheral neuropathy, acute renal failure (unresponsive to up to 72 h of rehydration, characterised by creatinine >500 μmol/L or urine output <400 mL per day, or requiring dialysis), or active or previous hepatitis C infection. The study was approved by the national ethics review board (National Research Ethics Service, London, UK), institutional review boards of the participating centres, and the competent regulatory authority (Medicines and Healthcare Products Regulatory Agency, London, UK), and was done according to the Declaration of Helsinki and the principles of Good Clinical Practice as espoused in the Medicines for Human Use (Clinical Trials) Regulations. The study was not originally designed as an adaptive trial however good recruitment and the emergence of data on novel combinations led to trial adaptation. These changes were proposed by the Trial Management Group and approved by the independent Data Monitoring and Ethics Committee and Trial Steering Committee. The final study protocol is in the appendix (p 19). All patients provided written informed consent.

### Randomisation and masking

Patients considered eligible for transplantation at trial entry were randomly assigned (1:1) to induction treatment with either cyclophosphamide, thalidomide, and dexamethasone (CTD) or cyclophosphamide, lenalidomide, and dexamethasone (CRD). A computer-generated minimisation algorithm with a random element was used to avoid chance imbalances in six variables measured at trial entry: β_2_-microglobulin (<3·5 mg/L *vs* 3·5–<5·5 mg/L *vs* ≥5·5 mg/L *vs* unknown), haemoglobin (<115 g/L *vs* ≥115 g/L for men; <95 g/L *vs* ≥95 g/L for women), corrected serum calcium (<2·6 mmol/L *vs* ≥2·6 mmol/L), serum creatinine (<140 μmol/L *vs* ≥140 μmol/L), platelets (<150 × 10^9^ cells per L *vs* ≥150 × 10^9^ cells per L), and centre (each centre is listed in the appendix p 15). Following a protocol amendment on June 28, 2013, and after the recruitment of 1512 patients, patients considered eligible for transplantation were randomly assigned (1:1:2) to CTD, CRD, or carfilzomib, cyclophosphamide, lenalidomide, and dexamethasone (KCRD). A similar minimisation algorithm with a random element was used to avoid chance imbalances in the six variables measured at trial entry. These changes were made to add a new induction treatment research question to this adaptive design study and were approved by the independent Data Monitoring and Ethics Committee and Trial Steering Committee. Patients considered ineligible for transplantation at trial entry were randomly assigned (1:1) to induction with either attenuated CTD or attenuated CRD. A similar minimisation algorithm with a random element was used to avoid chance imbalances in the six variables measured at trial entry.

Patients with a suboptimal response to induction treatment were randomly assigned (1:1) to cyclophosphamide, bortezomib, and dexamethasone (CVD) or no CVD. A minimisation algorithm with a random element was used to avoid chance imbalances in three variables: allocated induction treatment (CTD *vs* CRD *vs* attenuated CTD *vs* attenuated CRD), response to induction treatment (minimal or partial response), and centre. Patients allocated to KCRD induction treatment were ineligible for this randomisation.

Patients completing induction and intensification treatment (where applicable) and eligible were randomly assigned (1:1) to lenalidomide maintenance or observation. A minimisation algorithm with a random element was used to avoid chance imbalances in three variables: allocated induction treatment (CTD *vs* CRD *vs* attenuated CTD *vs* attenuated CRD), allocated intensification treatment (no CVD *vs* CVD *vs* not randomly assigned at intensification randomisation), and centre. Following a protocol amendment on Sep 14, 2011, and after recruitment of 442 patients under protocol versions 2.0 to 4.0, patients were randomly assigned (1:1:1) to lenalidomide, lenalidomide plus vorinostat, or observation. A similar minimisation algorithm with a random element was used to avoid chance imbalances in the same three variables with the same categories. Following a protocol amendment on June 28, 2013, and after recruitment of 615 further patients under protocol version 5.0, patients were randomly assigned (2:1) to lenalidomide or observation. A similar minimisation algorithm with a random element was used to avoid chance imbalances in the same three variables with the same categories but with the addition of KCRD to the induction treatment options. These changes were made to add and remove research questions in maintenance in this adaptive design study and were approved by the independent Data Monitoring and Ethics Committee and Trial Steering Committee.

All randomisations were done at the Clinical Trials Research Unit (Leeds, UK) by authorised members of staff with a centralised automated 24-h telephone system according to a validated minimisation algorithm produced under the supervision of WMG. Because of the nature of the intervention, the study was open label and the allocated treatment was not masked from study investigators or patients. The funders remained masked to treatment results until data cutoff.

### Procedures

The dose, schedule, and route of administration of each drug included in the induction, intensification, and maintenance regimens are described in the appendix (p 3). Briefly, in transplantation-eligible patients, induction therapy with CTD, CRD, or KCRD continued for at least four cycles in the absence of progressive disease, until maximum response or intolerance was observed. In transplantation-ineligible patients, attenuated CTD or attenuated CRD continued for at least six cycles in the absence of progressive disease, until maximum response or intolerance was observed. Attenuated versions of induction included lower doses of dexamethasone and a lower starting dose of thalidomide. For all patients, bisphosphonates were recommended until progressive disease and thromboprophylaxis was recommended for at least the first 3 months of treatment as per International Myeloma Working Group recommendations. Growth factor support and prophylaxis for pneumonia, varicella, fungal infection, and tumour lysis syndrome were allowed as per local practice.

Transplantation-eligible patients receiving KCRD proceeded to high-dose melphalan and autologous haemopoietic stem cell transplantation. Patients receiving immunomodulatory-based triplets (CTD *vs* CRD) followed a response-adapted approach with induction treatment intensification: those with complete response or very good partial response (assessed according to International Myeloma Working Group criteria) proceeded to transplantation in the transplantation-eligible pathway, whereas transplantation-ineligible patients proceeded directly to maintenance randomisation.

Transplantation-eligible and transplantation-ineligible patients allocated to induction treatment intensification received cyclophosphamide (500 mg daily orally on days 1, 8, and 15), bortezomib (1·3 mg/m^2^ subcutaneously or intravenously on days 1, 4, 8, and 11), and dexamethasone (20 mg daily orally on days 1, 2, 4, 5, 8, 9, 11, and 12) up to a maximum of eight cycles of 21 days in the absence of progressive disease, until maximum response or intolerance was observed. Full details of the dose reduction schedules are shown in the appendix (p 4).

For maintenance therapy, 100 days after autologous haemopoietic stem cell transplantation or once a maximum response was achieved for transplantation-ineligible patients, patients initially received lenalidomide or were observed without lenalidomide therapy. Following a protocol amendment on Sep 14, 2011, and after recruitment of 442 patients, patients were allocated (1:1:1) to receive lenalidomide, lenalidomide plus vorinostat, or observation. After recruitment of 615 more patients, a further protocol amendment on June 28, 2013, allocated patients to receive lenalidomide or observation in a 2:1 ratio, and the lenalidomide plus vorinostat group was discontinued. Maintenance treatment continued until progressive disease in the absence of toxicity.

Response and disease progression were assessed on the basis of International Myeloma Working Group Uniform Response criteria^9^, ^10^, ^11^ and reviewed centrally by an expert panel masked to treatment allocation. Adverse events were graded according to the US National Cancer Institute Common Terminology Criteria for Adverse Events version 4.0.

Adverse reactions were assessed at the start of each treatment cycle in patients receiving induction intensification. Comparisons between randomised groups were not made as adverse reactions could not be collected for those allocated to no CVD. Serious adverse events were reported for all patients from the date of randomisation until 30 days after the date of disease progression except in the case of serious adverse reactions or second primary malignancies, which were collected for the duration of the trial. Comparisons of these events were made for induction and maintenance comparisons only. Paraprotein, serum-free light chain, and urinary light chain excretion were assessed at least every 2 months for the first 2 years and then at least every 3 months until disease progression.

Cytogenetic risk profiling was done by use of multiplex ligation-dependent probe amplification using DNA and real-time PCR (rtPCR) using RNA, which were extracted from CD138-selected plasma cells from bone marrow biopsy samples taken before treatment initiation. rtPCR was used to assess the expression of translocation gene partners including t(4;14):*MMSET, FGFR3*, t(14;16):*MAF*, and t(14;20):*MAFB*. Multiplex ligation-dependent probe amplification was used to assess copy number aberrations by including probe sets at sites of the commonly deleted or amplified regions in myeloma (eg, at genes *CKS1B* on 1q21.3 and *TP53* on 17p13). These techniques are validated and provide equivalent results to interphase fluorescence in-situ hybridisation.^12^, ^13^, ^14^ Patients were classified into three cytogenetic risk groups for the preplanned analysis of outcomes: standard risk (no adverse cytogenetic abnormalities), high risk (one adverse cytogenetic abnormality), or ultra-high risk (two or more adverse cytogenetic abnormalities). Adverse cytogenetic abnormalities were defined as gain(1q), t(4;14), t(14;16), t(14;20), or del(17p).^15^, ^16^

### Outcomes

The co-primary endpoints of the induction intensification evaluation of the trial were progression-free and overall survival. Progression-free survival was defined as the time from induction intensification randomisation to progressive disease or death from any cause. Overall survival was defined as the time from induction intensification to death from any cause or last follow-up.

Secondary endpoints were response (including the proportion of conversions from minimal or partial response to very good partial response or better in patients allocated to CVD), progression-free survival 2 (defined as the time from induction intensification randomisation to the date of second progressive disease, start of third antimyeloma treatment, or death from any cause), and toxicity.

Exploratory analyses of progression-free survival, overall survival, and response by cytogenetic risk group were prespecified in the protocol and by induction treatment were prespecified in the statistical analysis plan within each pathway. Other exploratory endpoints will be reported elsewhere.

### Statistical analyses

The data cutoff date for this analysis was Jul 28, 2016. The hypothesis of the induction intensification randomisation was that CVD treatment could improve progression-free survival and overall survival compared with no CVD in adult patients with newly multiple myeloma. The overall study includes PFS and overall survival as co-primary endpoints for each randomisation. However, for the intensification element of the study only PFS was powered.

For PFS in the transplantation-eligible pathway, the trial was designed to show a 9-month increase in median progression-free survival in the CVD group (median 35 months) compared with the no CVD group (median 26 months, hazard ratio [HR] 0·74) when a total of 361 progression-free survival events had been observed. This calculation assumed the time-to-event was exponentially distributed and that recruitment would last 4 years with a further 4 years of follow-up, a two-sided 5% significance level, and 80% power. A recruitment target of 476 patients randomly assigned (1:1) to CVD or no CVD in the transplantation-eligible pathway was specified, allowing for 5% dropout. For progression-free survival in the transplantation-ineligible pathway, the trial was designed to demonstrate a 6-month increase in median progression-free survival in the CVD group (median 14 months) compared with the no CVD group (median 20 months, HR 0·70) when 337 PFS events had been observed. This calculation was based on similar assumptions but with a two-sided 5% significance level and 90% power. A recruitment target of 380 patients randomly assigned (1:1) to CVD or no CVD in the transplantation-eligible pathway was specified, allowing for 5% dropout. No power calculations were specified for the overall survival endpoint and analysis was triggered by induction analysis. The trial was designed assuming 47% of patients would be eligible following suboptimal response in each treatment pathway. These assumptions and estimated outcomes without CVD were based on results from our previous study, Myeloma IX.^7^

Efficacy analyses were done by intention to treat, including all patients randomly assigned to either CVD or no CVD. The safety population included all patients who received at least one dose of CVD therapy or those assigned to no CVD. For the co-primary endpoints, we estimated summaries of time to event per treatment group using the Kaplan-Meier method. We made comparisons between the allocated groups using the Cox proportional hazards model adjusted for the minimisation stratification factors, excluding centre, and stratified by treatment pathway, to estimate HRs and 95% CIs. Subgroup analysis was prespecified for the presence or absence of individual adverse cytogenetic abnormalities, cytogenetic risk status, and induction treatment. We did a likelihood ratio test for heterogeneity of treatment effect using Cox models identical to those used for the main analysis, with the inclusion of terms for the subgroup in question and the appropriate interaction terms. The reported test for heterogeneity for subgroup analysis corresponds to a one degree of freedom test for two category subgroups and a two degrees of freedom test for three category subgroups, etc. The number and proportion of participants in each response category was summarised descriptively and exact 95% CIs calculated using the Clopper-Pearson method. We summarised toxicity, in terms of adverse events, descriptively.

Post-hoc exploratory analyses were the effect of CVD on progression-free survival and overall survival within the subgroups sex, age, and disease stage according to the International Staging System. The proportional hazards assumptions were assessed by plotting the hazards over time (ie, the log cumulative hazard plot) for each treatment group, the methods described by Lin and colleagues^17^ were used to check the adequacy of the Cox regression model. Evidence was found to support a violation of the proportional hazards assumption in the progression-free survival comparison. Post-hoc exploratory analysis using restricted mean survival time methods^18^ was used to compare PFS times in the transplantation-eligible pathway. The parameter t* (ie, the area under the survival curve up to a time horizon) in the restricted mean survival time estimation, the expected progression-free period in this study, was chosen to be 64 months, because this was the maximum follow-up for all patients in the study. Other values of t* were investigated for the transplantation-ineligible pathway.

Formal interim analyses were prespecified in the study protocol for response and progression-free survival. A formal interim analysis was prespecified in the study protocol to ensure at least a 15% conversion of partial and minimal response patients to complete or very good partial response. The number of conversions was reviewed by the Myeloma XI data monitoring and ethics committee after one response in 19 patients who had completed CVD treatment, three in 50, and 22 in 200, with prespecified stopping criteria determined using exact probabilities for a Gehan two-stage design with 95% power.^19^ Additionally, at the last of these interim analyses, the possibility of an exceptionally large early effect of CVD on outcomes was evaluated using prespecified criteria of a 50% conversion or an increase in median progression-free survival of 24 months compared with no CVD (45 *vs* 21 months; HR 0·47). To ensure an overall significance level of 0·05 was maintained, the O’Brien and Fleming alpha-spending function was used with prespecified bounds of 0·005 for interim and 0·047 for final analysis.^20^ The interim analysis was done and presented to the data monitoring and ethics committee on Nov 14, 2014, and the study continued without reporting the interim analysis. All reported p values are two sided and considered significant at an overall significance level of 5%.

We used SAS (version 9.4), Stata/IC (version 14.2), and R (version 3.2.3) for statistical analyses. This study is registered with the ISRCTN registry, number ISRCTN49407852, and clinicaltrialsregister.eu, number 2009-010956-93.

### Role of the funding source

The funder of the study had no role in study design, data collection, data analysis, data interpretation, or writing of the report. The corresponding author had full access to all the data in the study and had final responsibility for the decision to submit for publication.

## Results

Of 3894 patients enrolled in the triplet induction element of the trial, 1217 achieved partial response or minimal response after induction therapy, of whom 583 (48%) were randomly assigned to CVD or no CVD in the intensification randomisation. Reasons for exclusion before intensification randomisation included withdrawn consent (n=302), ineligibility (n=37), proceeded directly to autologous haemopoietic stem cell transplantation (n=62), proceeded directly to maintenance therapy (n=25), progressive disease (n=78), and death (n=130). Slightly higher attrition occurred in the transplantation-ineligible pathway than transplantation-eligible but no difference in attrition between patients treated with lenalidomide or thalidomide-based induction was observed. The number of patients achieving partial or minimal response to each induction triplet entering the CVD randomisation were 200 (60%) of 333 in the CTD group, 167 (61%) of 276 in the CRD group, 118 (34%) of 345 in the attenuated CTD group, and 98 (37%) of 263 in the attenuated CRD group.

The induction intensification randomisation occurred between Nov 15, 2010, and Jul 28, 2016. 289 patients were assigned to receive CVD and 294 patients were assigned to no CVD (figure 1), of which 367 were enrolled in the transplantation-eligible pathway and 216 were enrolled in the transplantation-ineligible pathways. Patient and disease characteristics were well balanced between groups (table 1). The median time from induction therapy randomisation to CVD randomisation was 5·7 months (IQR 4·7–6·9) in the CVD group versus 5·8 months (4·6–6·7) in the no CVD group.

**Table 1:** Baseline characteristics

		**CVD group, N=289**	**No CVD group, N=294**
Age, years		66·0 (57·0–72·0)	66·0 (57·0–72·0)
Age group			
	≤65 years	147 (51%)	152 (52%)
	>65 years	142 (49%)	142 (48%)
Sex			
	Male	175 (61%)	158 (54%)
	Female	114 (39%)	136 (46%)
Ethnicity			
	White	271 (94%)	263 (89%)
	Black (eg, Black Caribbean, Black African)	5 (2%)	10 (3%)
	Asian (eg, Indian, Pakistani, Bangladeshi)	4 (1%)	7 (2%)
	Other	5 (2%)	3 (1%)
	Unknown	4 (1%)	11 (4%)
International Staging System			
	I	95 (33%)	90 (31%)
	II	129 (45%)	126 (43%)
	III	51 (18%)	60 (20%)
	Unknown	14 (5%)	18 (6%)
Immunoglobulin subtype			
	IgG	228 (79%)	219 (74%)
	IgA	46 (16%)	57 (19%)
	IgM	0 (0%)	0 (0%)
	IgD	0 (0%)	2 (<1%)
	Light chain only	15 (5%)	15 (5%)
	Non-secretor	0 (0%)	1 (<1%)
Cytogenetic risk available		129 (45%)	131 (45%)
Cytogenetic risk*			
	Standard	79 (61%)	71 (54%)
	High	32 (25%)	48 (37%)
	Ultra-high	18 (14%)	12 (9%)
Creatinine, μmol/L		84·0 (71·0–100·0)	84·0 (70·0–104·0)
	Unknown	0 (0%)	1 (<1%)
Transplantation eligibility			
	Transplantation eligible	183 (63%)	184 (63%)
	Transplantation ineligible	106 (37%)	110 (37%)
Induction therapy			
	CRD	85 (29%)	82 (28%)
	CTD	98 (34%)	102 (35%)
	CRD attenuated dose	49 (17%)	49 (17%)
	CTD attenuated dose	57 (20%)	61 (21%)
Time from induction randomisation to CVD randomisation, months		5·7 (4·7–6·9)	5·8 (4·6–6·7)

Data are median (IQR) or n (%). C=cyclophosphamide. D=dexamethasone. R=lenalidomide. T=thalidomide. V=bortezomib.

* High risk was defined as gain(1q), t(4;14), t(14;16), t(14;20), or del(17p), and ultra-high risk was defined as having two or more of these abnormalities. Percentages calculated as percentage of risk available.

**Figure 2:**
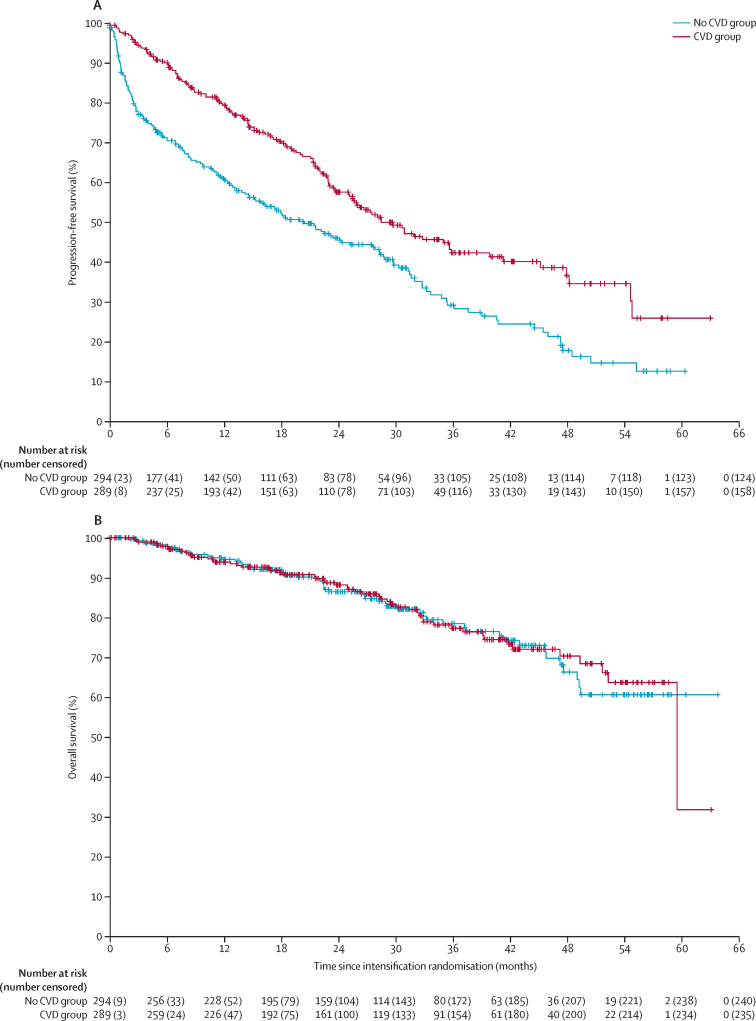
Progression-free and overall survival in the intention-to-treat population (A) Progression-free survival. (B) Overall survival. CVD=cyclophosphamide, bortezomib, and dexamethasone.

Median follow-up after randomisation was 29·7 months (IQR 17·0–43·5): 31·0 months (IQR 19·2–45·5) for transplantation-eligible patients and 27·3 months (IQR 14·7–40·9) for transplantation-ineligible patients. For the primary analyses, 131 (45%) of 289 patients in the CVD group and 170 (58%) of 294 patients in the no CVD group had disease progression or died. Median progression-free survival was 30 months (95% CI 25–36) in the CVD group and 20 months (15–28) in the no CVD group (HR 0·60, 95% CI 0·48–0·75, p<0·0001; figure 2A). Similar results were seen in the transplantation-eligible pathway: median progression-free survival was 48 months (95% CI 35–not reached) in the CVD group and 28 months (22–33) in the no CVD group (HR 0·50, 95% CI 0·36–0·68, p<0·0001; appendix p 9); and the transplantation-ineligible pathway: median progression-free survival was 20 months (95% CI 15–23·7) in the CVD group and 9 months (6–15) in the no CVD group (HR 0·73, 95% CI 0·52–1·02, p=0·061; appendix p 9).

There was some evidence that the proportional hazards assumption was violated overall (p<0·0001) and in the transplantation-eligible (p=0·059) and transplantation-ineligible pathway (p=0·0010), suggesting the treatment effect was not consistent over time. Outcomes were, therefore, further investigated using the restricted mean survival time method. This confirmed a significant benefit of CVD over no CVD in the overall population and in the transplantation-eligible pathway. This analysis further suggested a significant early benefit for CVD in the transplantation-ineligible pathway, which was lost at later time points (full analysis is presented in the appendix p 2).

54 (18%) of 289 patients died in the CVD group and 54 (19%) of 294 patients in the no CVD group. Median overall survival was not reached in either group. 3-year overall survival was 77·3% (95% CI 71·0–83·5) in the CVD group and 78·5% (72·3–84·6) in the no CVD group. No difference was detected between CVD and no CVD for overall survival (HR 0·98, 95% CI 0·67–1·43, p=0·93; figure 2B). The most common cause of death was tumour load (appendix p 5). Similar results were seen in the transplantation-eligible pathway: median overall survival was not reached in the CVD group and not reached in the no CVD group and 3-year overall survival was 81·5% (95% CI 74·4%–88·6%) in the CVD group and 81·2% (74·4%–87·9%) in the no CVD group (HR 0·83, 95% CI 0·50–1·36, p=0·45; appendix p 10); and the transplantation-ineligible pathway: median overall survival was not reached in the CVD group and not reached in the no CVD group and 3-year overall survival was 77·3% (95% CI 71·0–83·5) in the CVD group and 78·5% (72·3–84·6) in the no CVD group (HR 1·26, 95% CI 0·70–2·27, p=0·44; appendix p 10).

78 (27%) of 289 patients in the CVD group and 88 (30%) of 294 patients in the no CVD group had second disease progression or died. Median progression-free survival 2 was 52 months (95% CI 49–56) with CVD and 48 months (37–not reached) with no CVD (HR 0·83, 95% CI 0·61–1·22, p=0·22; appendix p 11). Similar results were seen in the transplantation-eligible pathway: median progression-free survival 2 was not reached with CVD and 53 months (49–not reached) with no CVD (HR 0·91, 95% CI 0·46–1·04, p=0·077; appendix p 11) and the transplantation-ineligible pathway: median progression-free survival 2 was 42 months (33–56 months) with CVD and 37 months (30–49) with no CVD (HR 1·06, 95% CI 0·66–1·70, p=0·81; appendix p 11).

Of the 289 patients randomly assigned to CVD, 243 (84%) had partial response and 24 (8%) had minimal response at the time of randomisation (at central review, 1 [<1%] had complete response, 12 [4%] had very good partial response, and 3 [1%] had stable or progressive disease); a similar distribution of responses was seen in the no CVD group (table 2). After CVD, 123 (42·6%, 95% CI 36·8–48·4) of 289 patients had very good partial response or better, with ten (3·5%, 1·7–6·3) achieving complete response and 113 (39·1%, 33·4–44·9) achieving very good partial response (table 2). The proportion of patients achieving very good partial response or better after CVD was similar regardless of the induction regimen received (43 [44%] of 98 for CTD, 39 [46%] of 85 for CRD, 22 [39%] of 57 for attenuated CTD, and 19 [39%] of 49 for attenuated CRD; appendix p 6). Among transplantation-eligible patients, response rates improved after autologous haemopoietic stem cell transplantation in both those randomly assigned to CVD and no CVD, but the final depth of response was greater in patients who received pre-transplant CVD. In the CVD group, 19 (14·3%, 8·8–21·4) of 133 patients achieved a complete response after ASCT and 68 (51·1%, 42·3–59·9) achieved a very good partial response (table 2). In the no CVD group, seven (5·9%, 2·4–11·7) of 119 achieved a complete response and 40 (33·6%, 25·2–42·9) achieved a very good partial response (table 2).

**Table 2:** Response by central review

	**After induction**		**After intensification**	**After autologous stem cell transplantation**	
	CVD group, N=289	No CVD group, N=294	CVD group, N=289	CVD group, N=133	No CVD group, N=119
Very good partial response or better	13 (4%)	12 (4%)	123 (43%)	87 (65%)	47 (39%)
Complete response	1 (<1%)	0 (0%)	10 (3%)	19 (14%)	7 (6%)
Very good partial response	12 (4%)	12 (4%)	113 (39%)	68 (51%)	40 (34%)
Partial response	243 (84%)	247 (84%)	113 (39%)	35 (26%)	65 (55%)
Minimal response	24 (8%)	22 (7%)	4 (1%)	2 (2%)	2 (2%)
Stable disease	0 (0%)	2 (<1%)	0 (0%)	0 (0%)	0 (0%)
Progressive disease	3 (1%)	9 (3%)	11 (4%)	3 (2%)	2 (2%)
Unavailable*	6 (2%)	2 (<1%)	38 (13%)	6 (5%)	3 (3%)

Data are n (%). CVD=cyclophosphamide, bortezomib, and dexamethasone.

* After intensification, Unavailable included no CVD received (n=17), CVD continuing (n=6), and death within 60 days of starting CVD (n=1); after autologous stem cell transplantation, Unavailable included death within 100 days after melphalan (n=1).

Patients received a median of four (range 3–5) cycles of CVD. 187 (65%) of 289 patients stopped due to achieving maximum response, whereas 34 (12%) stated unacceptable toxicity as the only or contributory reason for stopping. 144 (50%) of 289 patients had a dose modification during CVD treatment with cyclophosphamide modified in 58 (20%), bortezomib in 115 (40%) and dexamethasone in 74 (26%). The most common adverse events reported in the CVD group were anaemia (201 [73%] of 275), peripheral sensory neuropathy (165 [60%]), thrombocytopenia (129 [47%]), constipation (99 [36%]), neutropenia (74 [27%]), and diarrhoea (57 [21%]; table 3). Most events were mild-to-moderate in severity, with small numbers of grade 3 or higher adverse events (table 3). Other grade 3 or higher adverse events of note were lung infections (13 [5%]). Serious adverse events occurred in 103 (37%) patients during CVD intensification (appendix p 7), andd 58 (28%) patients had a serious adverse event related to study treatment. The most common treatment-related serious adverse event was infection, accounting for 42 (59%) of the 71 serious adverse events in the CVD group.

**Table 3:** Adverse events

	**Grade 1–2**	**Grade 3**	**Grade 4**
**Haematological**			
Neutropenia	56 (20%)	15 (5%)	3 (1%)
Anaemia	193 (70%)	8 (3%)	0 (0%)
Thrombocytopenia	110 (40%)	13 (5%)	6 (2%)
**Infections**			
Upper respiratory infection	23 (8%)	2 (<1%)	0 (0%)
Lung infection	18 (7%)	12 (4%)	1 (<1%)
Other	12 (4%)	4 (1%)	0 (0%)
**Neurological**			
Peripheral sensory neuropathy	155 (56%)	9 (3%)	1 (<1%)
Peripheral motor neuropathy	44 (16%)	3 (1%)	0 (0%)
**Gastroenterological**			
Constipation	98 (36%)	1 (<1%)	0 (0%)
Diarrhoea	52 (19%)	5 (2%)	0 (0%)
Nausea	44 (16%)	0 (0%)	0 (0%)
**Other**			
Fatigue	83 (30%)	3 (1%)	0 (0%)
Lethargy	20 (7%)	1 (<1%)	0 (0%)
Back pain	32 (12%)	3 (1%)	0 (0%)
Oedema limbs	35 (13%)	0 (0%)	0 (0%)
Hypotension	17 (6%)	3 (1%)	0 (0%)
Dizziness	16 (6%)	4 (1%)	0 (0%)

Data are n (%). The table includes all grade 1–2 adverse reactions occurring in at least 10% of patients and all grade 3 or 4 adverse reactions occurring at least 1% of patients in the CVD group (N=275). No grade 5 adverse reactions occurred. Sites were asked to report adverse reactions for patients taking the CVD and serious adverse events in all patients. Serious adverse events are shown in the appendix (p 7). CVD=cyclophosphamide, bortezomib, and dexamethasone.

**Figure 3:**
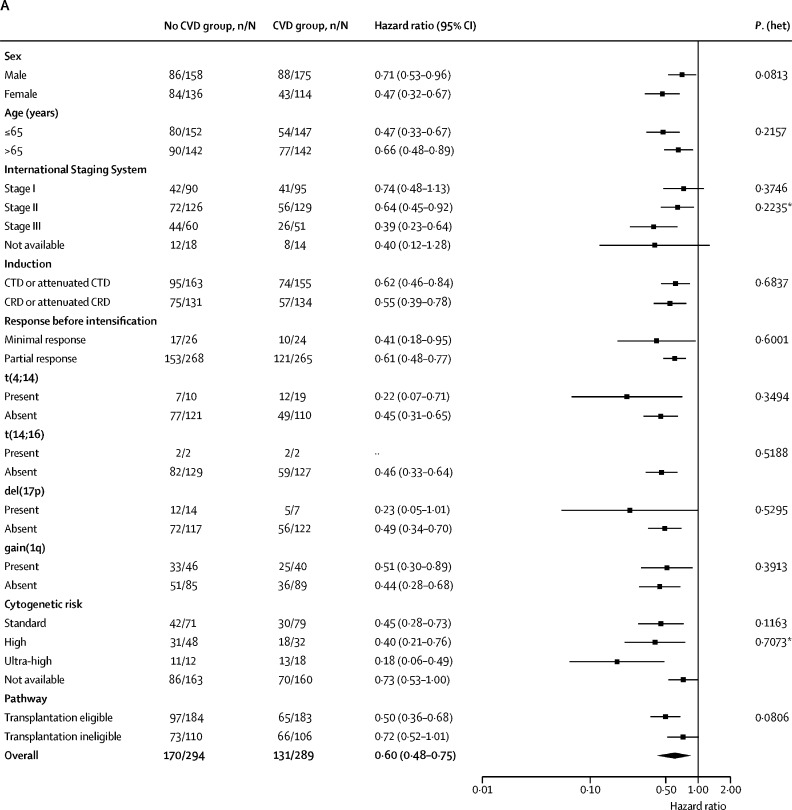

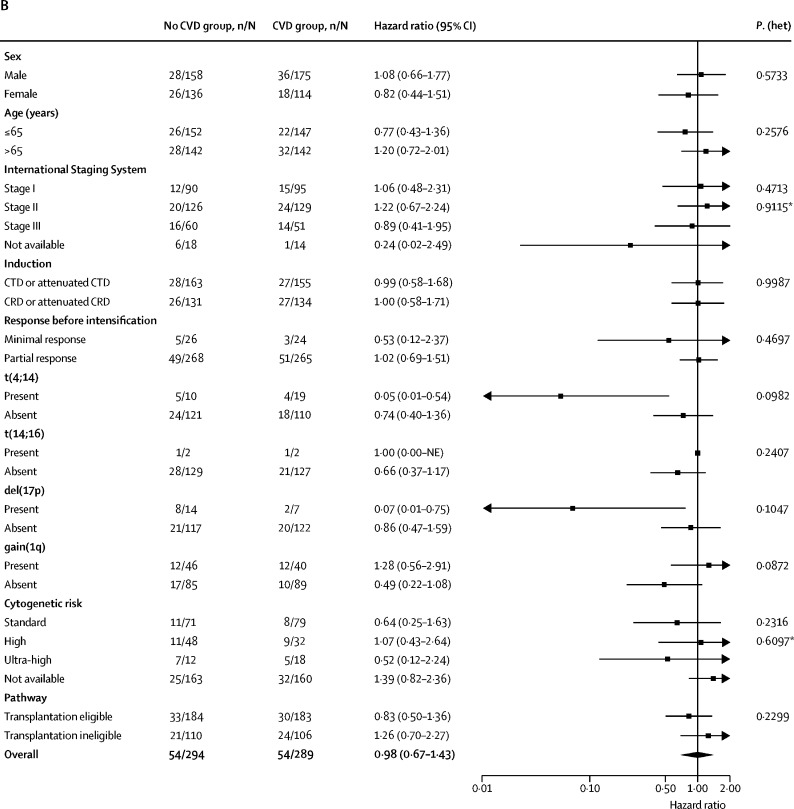
Subgroup analysis of progression-free and overall survival (A) Progression-free survival. (B) Overall survival. C=cyclophosphamide. D=dexamethasone. NE=Not estimable. R=lenalidomide. T=thalidomide. V=bortezomib. *Likelihood ratio test for heterogeneity of effect among patients with subgroup data available.

Subgroup analyses of progression-free survival and overall survival according to baseline characteristics were consistent with the results in the overall population (figure 3). In all subgroups, progression-free survival consistently favoured CVD versus no CVD irrespective of sex, age, induction therapy, and response at CVD randomisation. At initial randomisation, genetic risk had been evaluated in 260 (45%) of 583 patients (129 CVD; 131 no CVD). 150 (58%) of the 260 evaluable patients had standard risk, 80 (31%) had high risk, and 30 (12%) had ultra-high risk. The benefit of CVD versus no CVD in terms of progression-free survival was also seen across cytogenetic risk groups, including those with standard risk (HR 0·45, 95% CI 0·28–0·73), high risk (HR 0·40, 95% CI 0·21–0·76), and ultra-high risk (HR 0·18, 95% CI 0·06–0·49; appendix p 13) with no significant heterogeneity between subgroups (p_heterogeneity_=0·71) and numerically smaller HRs in the high risk and ultra-high risk cohorts, although the numbers of patients were small, particularly in the ultra-high risk cohort.

## Discussion

This study showed that additional therapy with CVD improved the depth of response for patients with newly diagnosed multiple myeloma with a suboptimal response to an immunomodulatory drug triplet combination, leading to marked improvements in progression-free survival compared with patients not receiving CVD intensification but without any difference in overall survival. The depth of response was very good partial response or better in 42·6% (95% CI 36·8–48·4) of patients treated with CVD. Median progression-free survival improved by 20 months in transplantation-eligible patients (HR 0·50, 95% CI 0·36–0·68) and 12 months in transplantation-ineligible patients (HR 0·73, 95% CI 0·52–1·02). Overall survival did not differ; however, median overall survival was not reached in either group at the time of analysis and after additional follow-up this endpoint will be reanalysed.

The absence of overall survival difference might have been confounded by the use of therapy after relapse, for example, which was not pre-specified. Outcomes in the no CVD group might also have been affected by subsequent treatment within the trial. Although progression free survival improved with lenalidomide versus observation in all patients in the maintenance randomisation, patients receiving no CVD appeared to have an even greater benefit than those who had CVD or were not randomly assigned (no CVD HR 0·26 [95% CI 0·17–0·40] *vs* CVD HR 0·48 [0·30–0·76] *vs* not randomly assigned HR 0·49 [0·43–0·57] p_heterogeneity_=0·011).^8^

The benefit of CVD therapy in terms of progression-free survival was consistent across subgroups and persisted after autologous haemopoietic stem cell transplantation in transplantation-eligible patients. The response rates after transplantation for the CVD and no CVD patients show that autologous haemopoietic stem cell transplantation alone can improve suboptimal initial response, but this can be significantly further improved by the use of pre-transplant intensification. The beneficial effect of CVD intensification on PFS appeared to be independent of cytogenetic risk status assessed centrally (with central results not fed back to local centres). These results are consistent with previous studies that suggest an additional benefit for proteasome inhibition particularly in the t(4;14) or del(17p) subsets of disease.^21^, ^22^

Statistically, evidence suggested that the hazard for not receiving CVD was not constant over time and, therefore, that the proportional hazards assumption was violated. This effect appeared more marked in the transplantation-ineligible pathway. Therefore, as prespecified in the statistical analysis plan, the restricted mean survival time method was used to confirm the results from the Cox regression analysis. This analysis confirmed the progression-free survival benefit associated with early exposure to CVD in transplantation-ineligible patients, possibly reflecting the effects of subsequent and later-line therapies in this patient subgroup.

From a biological perspective, our results are consistent with the concept that resistance to initial therapy is based on the specific therapy used. This resistance can be overcome by switching to a chemotherapy regimen with an alternate mechanism of action. We found that the adverse prognostic effect of poor initial response can be overcome and is not an inherent feature of the cancer itself but rather of the therapy administered. Analysis of patients within the trial achieving stable disease or progressive disease to immunomodulatory triplet induction who all received CVD intensification will explore this concept further and will be published separately. Additionally, in long-term follow-up, we plan to investigate the differences between achieving complete response or very good partial response to initial induction in contrast to only achieving it after CVD intensification. Minimal residual disease analysis was also done and this data will be presented separately.

In contrast to our use of induction intensification, two studies have examined the use of post-transplant consolidation. In the Stamina BMT CTN 0702 study,^23^ patients were treated with initial therapy at investigators discretion followed by randomisation before autologous haemopoietic stem cell transplantation to one of three strategies: transplantation followed by lenalidomide maintenance, two autologous stem cell transplants followed by lenalidomide maintenance, or transplantation followed by post-transplant consolidation with bortezomib, lenalidomide, and dexamethasone (VRD) and then by lenalidomide maintenance. No difference in progression-free survival or overall survival was identified between randomisation groups. In the EMN02 study,^24^ patients were enrolled before commencing treatment and treated with four cycles of CVD induction before being randomly assigned to autologous haemopoietic stem cell transplantation (one or two transplants depending on centre) or four cycles of bortezomib, melphalan, and prednisolone. There was a subsequent randomisation between two cycles of VRD or no consolidation. All patients received lenalidomide maintenance. In this study, post-transplant VRD consolidation before lenalidomide maintenance improved progression-free survival compared with maintenance alone (HR 0·78; p=0·045). The preliminary overall survival results indicated comparable 3-year overall survival with (86%) and without consolidation (87%). One of the key differences between studies was the initial induction therapy administered. In Stamina BMT CTN 0702, maximum response to induction therapy was more likely to have been achieved before autologous haemopoietic stem cell transplantation due to a lack of fixed duration of induction therapy. Similar to our study, patients had a median of 5 months (range 2–14 months) between initial therapy and registration and around 15% of patients had received more than one previous regimen, suggesting therapy changes in patients with a suboptimal initial response. In contrast, patients in EMN02 received a fixed four cycles of CVD given in 21-day cycles, equating to 3 months of therapy. Taken together, these two studies support the conclusions of our study and suggest that maximising treatment response, either before or after transplant, is an important endpoint of therapy.

One limitation of the Myeloma XI study might be the induction regimens used, CRD or CTD combinations followed by a proteasome-inhibitor-based intensification regimen. The trial was designed in this manner owing to the UK standard of care at the time with no access to bortezomib either alone or in combination with immunomodulatory drugs. However, the outcomes for patients in the Myeloma XI trial do not substantially differ from other novel combinations. In the IFM 2009 study,^25^ 700 transplantation-eligible patients were randomly assigned to either three cycles of VRD followed by autologous haemopoietic stem cell transplantation and two cycles of VRD or VRD for eight cycles without autologous haemopoietic stem cell transplantation. The study^25^ supported the use of upfront autologous haemopoietic stem cell transplantation, with the median progression-free survival being longer for patients who received RVD and transplantation compared with those who received RVD alone (50 *vs* 36 months, HR 0·65, 95% CI 0·53–0·80, p<0·001), although overall survival was similar in both treatment groups. The results obtained in Myeloma XI are not dissimilar. For example, the median progression-free survival from CVD randomisation (ie, not including the duration of previous induction cycles) was 48 months for those receiving CVD consolidation in Myeloma XI compared to 50 months from the start of induction for those who received VRD plus autologous haemopoietic stem cell transplantation in IFM 2009 (appendix p 8). VRD is not widely reimbursed outside of the USA, where the combinations used in this study remain pertinent. Furthermore, even in the setting of VRD, a proportion of patients do not respond well and so the findings of Myeloma XI would support personalising therapy by adding additional agents of a different class to induction in the absence of a deep initial response. About half of the patients in the Myeloma XI trial with partial or minimal response after induction therapy discontinued the study before intensification randomisation for various reasons and are, therefore, not included in the analysis presented here. These reasons included progressive disease and death, but also off-study treatment. Although further details for each individual patient withdrawing consent were not collected, we believe these withdrawals might have been driven by investigator and patient discomfort with delivering intensification to patients with responses very close to very good partial response but not quite meeting it or, conversely, not delivering intensification to patients with a response of only minimal response to initial induction. The substantial number of patients not reaching trial randomisation supports the use of combination therapies upfront to maximise response and improve outcomes, and these are now standard of care in the UK with the combination bortezomib, thalidomide, and dexamethasone.

Taken together our data suggest that, in the case of suboptimal response to initial treatment, agent class should be switched rapidly with the aim of response intensification. Although patients who do not respond to the induction therapy might still benefit from autologous haemopoietic stem cell transplantation,^26^ these data support the maximisation of response before transplantation, given that the depth of response is associated with improved outcomes.

## Data sharing

De-identified participant data will be made available when all primary and secondary endpoints have been met. Any requests for trial data and supporting material (data dictionary, protocol, and statistical-analysis plan) will be reviewed by the trial-management group in the first instance. Only requests that have a methodologically sound proposal and whose proposed use of the data has been approved by the independent trial steering committee will be considered. Proposals should be directed to the corresponding author in the first instance. To gain access, data requestors will need to sign a data access agreement.

## References

[1] Pawlyn C, Morgan GJ (2017). Evolutionary biology of high-risk multiple myeloma. Nat Rev Cancer.

[2] Morgan GJ, Walker BA, Davies FE (2012). The genetic architecture of multiple myeloma. Nat Rev Cancer.

[3] Lahuerta JJ, Paiva B, Vidriales MB (2017). Depth of response in multiple myeloma: a pooled analysis of three PETHEMA/GEM clinical trials. J Clin Oncol.

[4] Paiva B, van Dongen JJ, Orfao A (2015). New criteria for response assessment: role of minimal residual disease in multiple myeloma. Blood.

[5] Rawstron AC, Child JA, de Tute RM (2013). Minimal residual disease assessed by multiparameter flow cytometry in multiple myeloma: impact on outcome in the Medical Research Council Myeloma IX study. J Clin Oncol.

[6] Morgan GJ, Davies FE, Gregory WM (2011). Cyclophosphamide, thalidomide, and dexamethasone (CTD) as initial therapy for patients with multiple myeloma unsuitable for autologous transplantation. Blood.

[7] Morgan GJ, Davies FE, Gregory WM (2012). Cyclophosphamide, thalidomide, and dexamethasone as induction therapy for newly diagnosed multiple myeloma patients destined for autologous stem-cell transplantation: MRC Myeloma IX randomized trial results. Haematologica.

[8] Jackson GH, Davies FE, Pawlyn C (2019). Lenalidomide maintenance versus observation for patients with newly diagnosed multiple myeloma (Myeloma XI): a multicentre, open-label, randomised, phase 3 trial. Lancet Oncol.

[9] Bladé J, Samson D, Reece D (1998). Criteria for evaluating disease response and progression in patients with multiple myeloma treated by high-dose therapy and haemopoietic stem cell transplantation. Br J Haematol.

[10] Durie BG, Harousseau JL, Miguel JS (2006). International uniform response criteria for multiple myeloma. Leukemia.

[11] Rajkumar SV, Harousseau JL, Durie B (2011). Consensus recommendations for the uniform reporting of clinical trials: report of the International Myeloma Workshop Consensus Panel 1. Blood.

[12] Shah V, Sherborne AL, Walker BA (2018). Prediction of outcome in newly diagnosed myeloma: a meta-analysis of the molecular profiles of 1905 trial patients. Leukemia.

[13] Boyle EM, Proszek PZ, Kaiser MF (2015). A molecular diagnostic approach able to detect the recurrent genetic prognostic factors typical of presenting myeloma. Genes Chromosomes Cancer.

[14] Kaiser MF, Walker BA, Hockley SL (2013). A TC classification-based predictor for multiple myeloma using multiplexed real-time quantitative PCR. Leukemia.

[15] Boyd KD, Ross FM, Chiecchio L (2012). A novel prognostic model in myeloma based on co-segregating adverse FISH lesions and the ISS: analysis of patients treated in the MRC Myeloma IX trial. Leukemia.

[16] Sonneveld P, Avet-Loiseau H, Lonial S (2016). Treatment of multiple myeloma with high-risk cytogenetics: a consensus of the International Myeloma Working Group. Blood.

[17] Lin DY, Wei LJ, Ying Z (1993). Checking the Cox model with cumulative sums of Martingale-based residuals. Biometrika.

[18] Royston P, Parmar MKB (2011). The use of restricted mean survival time to estimate the treatment effect in randomized clinical trials when the proportional hazards assumption is in doubt. Statist Med.

[19] Gehan EA (1961). The determination of number of patients required in a preliminary and a follow-up trial of a new chemotherapeutic agent. J Chronic Dis.

[20] O'Brien PC, Fleming TR (1979). A multiple testing procedure for clinical trials. Biometrics.

[21] Avet-Loiseau H, Leleu X, Roussel M (2010). Bortezomib plus dexamethasone induction improves outcome of patients with t(4;14) myeloma but not outcome of patients with del(17p). J Clin Oncol.

[22] Sonneveld P, Goldschmidt H, Rosinol L (2013). Bortezomib-based versus nonbortezomib-based induction treatment before autologous stem-cell transplantation in patients with previously untreated multiple myeloma: a meta-analysis of phase III randomized, controlled trials. J Clin Oncol.

[23] Stadtmauer EA, Pasquini MC, Blackwell B (2019). Autologous transplantation, consolidation, and maintenance therapy in multiple myeloma: results of the BMT CTN 0702 trial. J Clin Oncol.

[24] Sonneveld P, Beksac M, van der Holt B (2016). Consolidation followed by maintenance therapy versus maintenance alone in newly diagnosed, transplant eligible patients with multiple myeloma (MM): a randomized phase 3 study of the European Myeloma Network (EMN02/HO95 MM trial). Blood.

[25] Attal M, Lauwers-Cances V, Hulin C (2017). Lenalidomide, bortezomib, and dexamethasone with transplantation for myeloma. N Engl J Med.

[26] Kumar S, Lacy MQ, Dispenzieri A (2004). High-dose therapy and autologous stem cell transplantation for multiple myeloma poorly responsive to initial therapy. Bone Marrow Transplant.

